# The Burden of Suicide Mortality in Poland: A 20-Year Register-Based Study (2000–2019)

**DOI:** 10.3389/ijph.2023.1605621

**Published:** 2023-02-02

**Authors:** Małgorzata Pikala, Monika Burzyńska

**Affiliations:** Department of Epidemiology and Biostatistics, Social and Preventive Medicine, Medical University of Lodz, Łódź, Poland

**Keywords:** Poland, epidemiology, suicide, mortality trends, hanging

## Abstract

**Objectives:** The aim of the study was to assess mortality trends due to suicide in Poland in the years 2000–2019 with the use of joinpoint regression.

**Methods:** The study analysed all suicide deaths in Poland in the years 2000–2019 (N = 113,355). Age-standardised death rates (SDRs), the annual percentage change (APC) and the average annual percentage change (AAPC) were determined.

**Results:** In the male group, SDR was 29.3 in 2000 and 21.6 in 2019, in the female group, SDR decreased from 5.2 to 3.0. In 2019, the highest SDR values were noted in the group aged between 45 and 64 years. The most common method of suicide was hanging. In 2019, odds ratios (OR) of death due to suicide for age groups 15–24 years vs. 65 years or above were 51.47 among men and 181.89 among women. With regards to primary vs. tertiary education, OR values were 1.08 and 0.25, respectively; for single vs. widowed individuals 8.22 and 12.35; while for rural vs. urban residents 1.60 and 1.15.

**Conclusion:** There is a need to implement educational programmes, primarily designed for young people.

## Introduction

In 2019, it was estimated that 703,000 people committed suicide worldwide [1]. Due to the stigma associated with suicide and the fact that this act is illegal in some countries, this number is underestimated since some suicides are classified as unintentional injuries [2].

Deaths from suicide account for approximately 1.3% of all deaths worldwide. In 2019, the worldwide suicide-related standardised death rate was 9.0 per 100,000 population.

Downward trends in mortality due to suicide are observed in most countries. Over the period of 20 years, between 2000 and 2019, the global age-standardised suicide rate decreased by 36% [1].

There is a very large variation in suicide mortality across Europe. According to Eurostat, in 2018 the highest death rates were observed in Lithuania (24.0), Slovenia (16.9) and Latvia (15.6), whereas the lowest ones were noted in Greece (4.9), Cyprus (4.6) and Turkey (4.5) [3].

Poland is a country with a population exceeding 38 million people. It is located in Central Europe, a geographical region consisting mainly of post-communist countries. After 1989, Poland, like other countries in this group, began to face numerous health challenges that emerged as a result of the transition from a centrally-planned economy to a market economy. The privatisation of production was accompanied by all kinds of adaptation problems - increased unemployment, social disparities in income, higher alcohol consumption [4]. A rapid transformation from state-controlled media to liberal media resulted in the promotion and adoption of some unhealthy habits of the Western lifestyle by the Polish society [5]. These changes affected suicide rates [6].

Death statistics recorded up to 1989 in Poland are unreliable as communist authorities kept suicide data secret [7]. The year 1989, being the beginning of changes related to the fall of communism, resulted in a decrease in suicide rates. The 1990s in Poland were marked by increased unemployment, poverty in certain social groups and a lack of hope for improvement. This was followed by disappointment with effects of the transformation accompanied by rising suicide death rates. Between 1989 and 1994, the suicide rate increased in Poland by 26.5% [4].

In Poland, as in other Eastern European countries, the value of the standardised death rate due to suicide is still quite high, and in 2019 it was 11.9. Poland had the 13th highest death rate due to suicide among EU countries. In many countries, the number of suicides exceeds the number of fatalities in road accidents and this also applies to Poland [8].

Suicide is one of the leading causes of death among young people. In the age group 15–29 years, suicides are the second most common cause of death, following road injuries. Approximately 200,000 adolescents and young adults commit suicides worldwide each year [9]. Deaths by suicide also occur in children aged 5–14 years. International reported suicide rates per 100,000 in this young population vary between 3.1 and 0 (the mean worldwide rate, approximately 0.6/100,000) [10]. Suicide is often committed by people at a young age, so it contributes to a large number of years of life lost. A study conducted in 2011 in Poland revealed that suicide was the second most important cause of the number of standard expected years of life lost per death after road accidents [11]. Lost years of life are associated with a social and economic loss. There are over a dozen methods in the world for calculating the cost of suicide for a state budget. In Poland, the so-called lost productivity method is used. The total cost of lost productivity due to suicide-related premature mortality among adolescents in 2019 was 170,100,369 PLN [12].

Furthermore, suicide evokes great pain for the victims’ relatives and for other survivors. The World Health Organization estimates that six people are closely affected by one suicide [13].

Social and economic costs of suicidal behaviour for individuals, their families and society make suicide a serious problem for both individual and public health. Suicide is largely preventable. Reduction of suicide mortality has become a priority for the World Health Organization, a global target included in the United Nations Sustainable Development Goals, i.e., the WHO 13th General Programme of Work 2019–2023 and the WHO Mental Health Action Plan 2013–2030 [1].

Implementation of preventive measures should be preceded by an accurate epidemiological diagnosis so that interventions are tailored to trends and needs of particular social groups.

The aim of this study was to assess trends in suicide-related mortality in Poland in the years 2000–2019 in gender and age groups. The odds of dying by committing suicide in socio-demographic groups and the frequency of specific methods of committing suicide were also analysed.

## Methods

### Database

The database used in the study was compiled from all death records of Polish residents issued between 2000 and 2019, obtained from the Statistics Poland. During the analysed 20 years, 7,610,613 people died in Poland. Of that number, 113,355 deaths (1.5%) were caused by suicide (according to the International Statistical Classification of Diseases and Related Health Problems—Tenth Revision—ICD-10, codes: X60-X84).

The death certificate includes the following variables: sex, age of the deceased, cause of death, education level, marital status, employment status and place of residence.

### Statistical Analysis

Standardised death rates (SDR) were calculated in the groups by sex and age. The standardisation procedure was performed with the use of the direct method, in compliance with the European Standard Population, updated in 2012 [14]. Separate analyses were conducted for the following age-groups: 5–14, 15–24, 25–44, 45–64 and 65+ years.

The analysis of time trends was carried out with the use of joinpoint models and the Joinpoint Regression programme, a statistical software package developed by the U.S. National Cancer Institute for the Surveillance, Epidemiology and End Results Program [15].

This method is an advanced version of linear regression, where time trend is expressed with a broken line, which is a sequence of segments joined in joinpoints. In these points, the change of the value is statistically significant (*p* < 0.05). We have also calculated annual percentage change (APC) for each segment of broken lines and average annual percentage change (AAPC) for the whole study period with corresponding 95% confidence intervals (CI). A logistic regression analysis was performed to identify sociodemographic factors (age, educational level, marital status, employment status and place of residence) that can contribute to suicide. The results were shown as odds ratios (OR) with 95% confidence intervals (CI). Statistical analyses were performed using the STATISTICA for Windows XP programme, version 13 (TIBCO Software Inc.).

## Results

### Temporal Trends in Suicide Rates

Over 20 years, between 2000 and 2019, 113,355 people died as a result of committing suicide in Poland. Of this number, 97,254 were men and 16,101 women (Table 1). The number of deaths as well as the standardised death rates (SDR) due to suicide between 2000 and 2019 decreased in both gender groups.

**TABLE 1:** Characteristics of people who died due to suicide according to sociodemographic variables, Poland, 2000–2019.

	Men		Women	
	Number of deaths N = 97,254	%	Number of deaths N = 16,101	%
Age group				
5–14	264	0.27	101	0.63
15–24	9,968	10.25	1,358	8.43
25–44	32,043	32.95	3,673	22.81
45–64	41,233	42.40	7,127	44.26
65+	13,746	14.13	3,842	23.87
Educational level^a^				
Elementary	32,278	33.79	5,752	36.21
Secondary	58,757	61.51	8,500	53.50
University	4,494	4.70	1,635	10.29
Marital status^b^				
Single	34,328	35.69	3,255	20.42
Married	44,344	46.10	7,281	45.67
Divorced	12,105	12.58	2,181	13.68
Widowed	5,419	5.63	3,227	20.23
Employment status^c^				
economically active	22,074	23.64	2,476	15.97
economically inactive	71,299	76.36	13,024	84.03
Place of residence				
Rural	48,977	50.36	9,491	58.95
Urban	48,277	49.64	6,610	41.05

^a^ In 1939 death certificates (1.71%), there was no data on educational level.

^b^ In 1215 death certificates (1.07%), there was no data on marital status.

^c^ Data on the employment status refer to the years 2000–2014. Since 2015, information on the employment status has been removed from death cards in Poland. In 1154 death certificates (1.21%), there was no data on the employment status.

**FIGURE 1:**
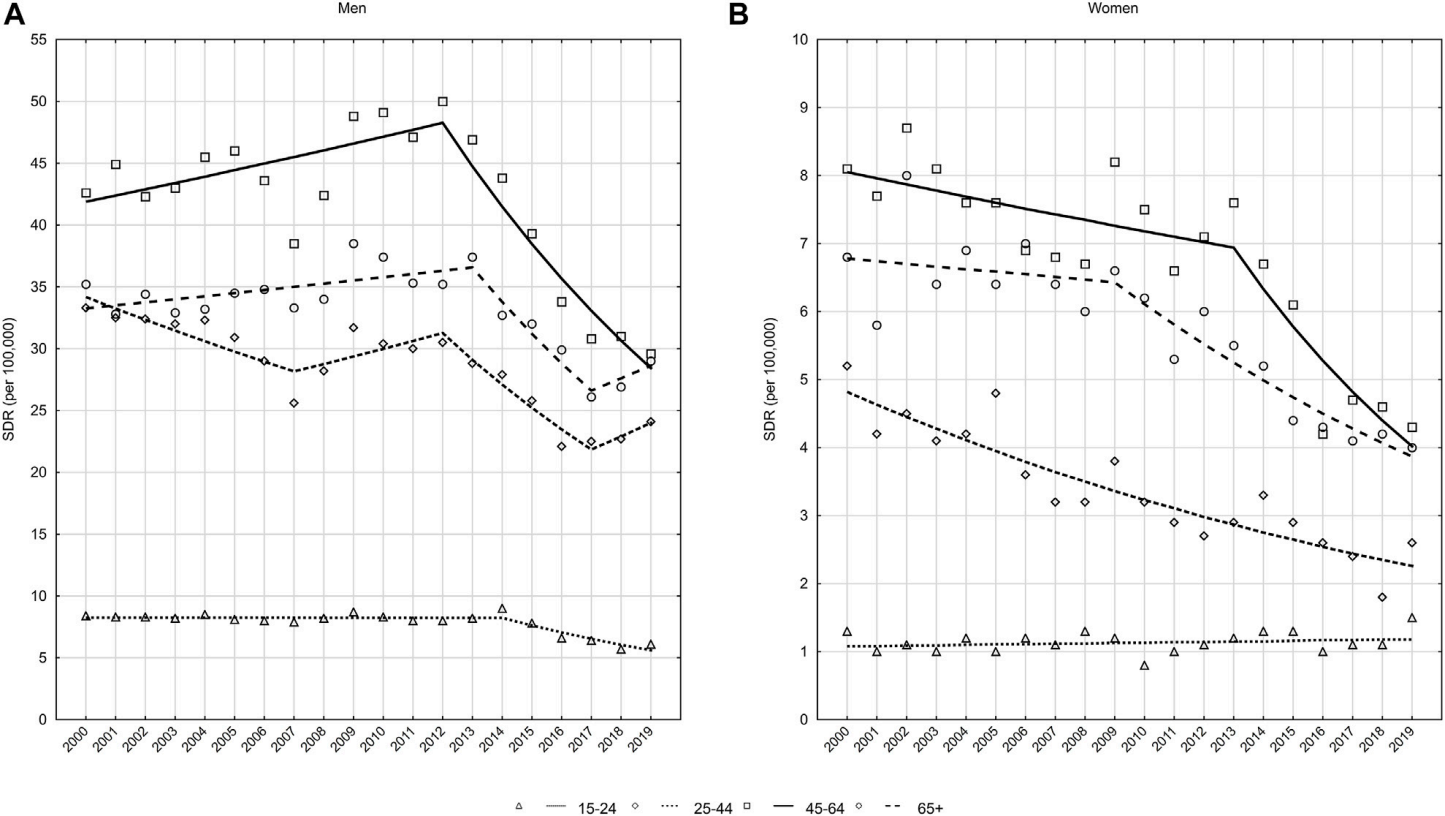
Suicide, number of deaths and standardized mortality rates (SDR) by sex, Poland, 2000–2019.

**FIGURE 2:**
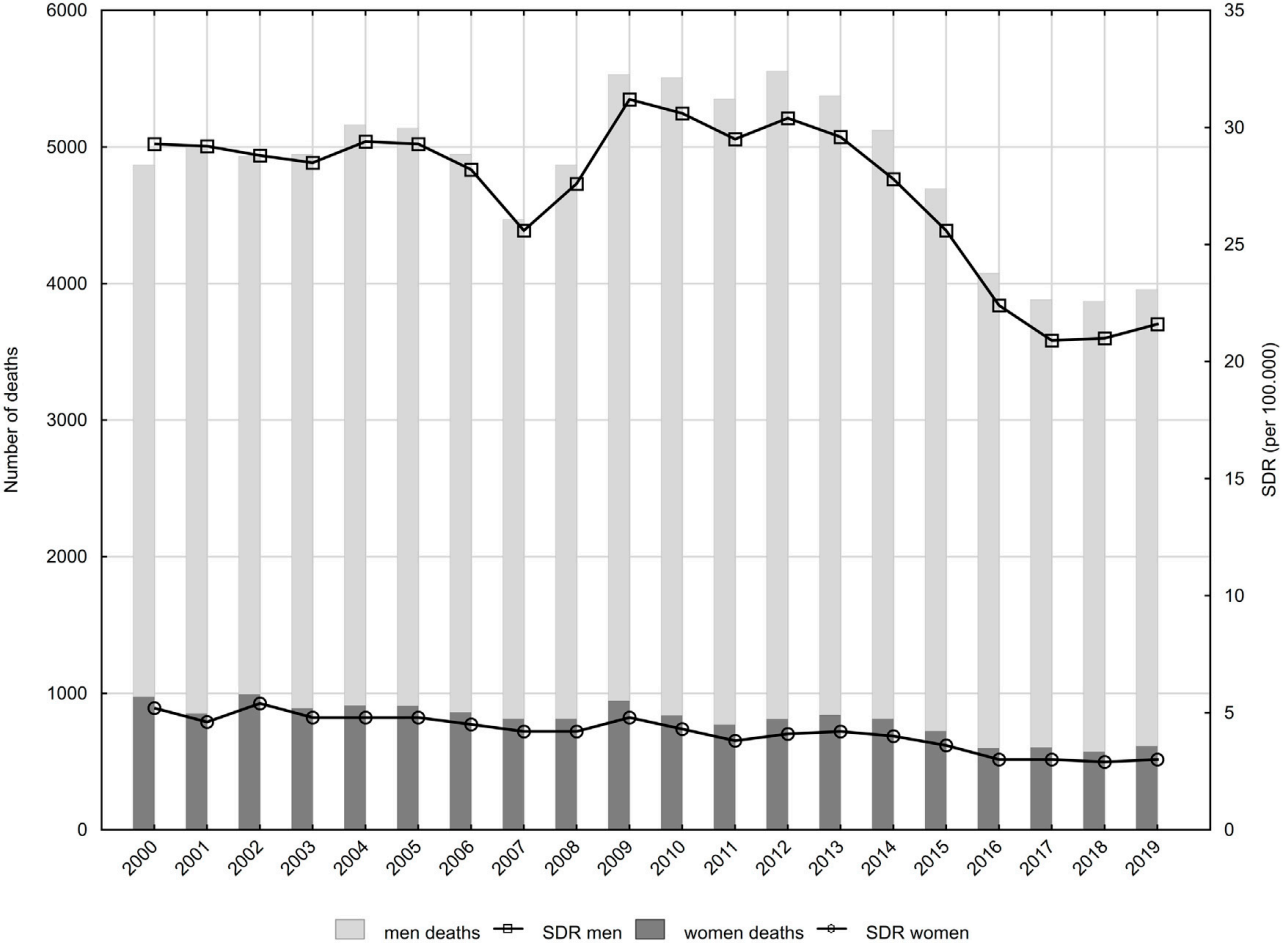
Suicide, time trends of standardised death rates (SDR) by gender and age groups, Poland, 2000–2019.

In the male group, there were 4,869 deaths by suicide in 2000, which represented 29.3 deaths per 100,000 men. In 2019, the number of deaths decreased to 3,957 and SDR was 21.6 (Figure 1). The average annual percentage change (AAPC) between 2000 and 2019 was −1.5 and a statistically significant decrease was observed only between 2013 and 2017, when the annual percentage change (APC) was −8.5%. There was a statistically insignificant increase of 0.4% and 1.5% in the periods 2000–2013 and 2017–2019, respectively (Table 2) (Figure 2).

**TABLE 2:** Suicide, time trends of standardised death rates by gender and age groups—joinpoint regression analysis, Poland, 2000–2019.

Age group	Number of joinpoints	Years	APC (95% CI)	AAPC (95% CI)
Men				
0+	2	2000–2013	0.4 (−0.4; 1.2)	−1.5 (−3.5; 0.6)
		2013–2017	−8.5* (−15.2; −1.3)	
		2017–2019	1.5 (−12.9; 18.1)	
5–14	0	2000–2019	−5.1* (−7.5; −2.6)	
15–24	1	2000–2014	0.0 (−0.6; 0.5)	−2.0* (−2.7; −1.3)
		2014–2019	−7.4* (−9.8; −4.9)	
25–44	3	2000–2007	−2.7* (−4.3; −1.1)	−1.8* (−3.5; −0.1)
		2007–2012	2.1 (−1.7; 6.1)	
		2012–2017	−6.9* (−10.4; −3.3)	
		2017–2019	4.8 (−7.1; 18.2)	
45–64	1	2000–2012	1.2* (0.1; 2.3)	−2.0* (−3.0; −1.0)
		2012–2019	−7.3* (−9.6; −5.0)	
65+	2	2000–2013	0.7* (0.1; 1.4)	−0.8 (−2.5; 0.9)
		2013–2017	−7.6* (−13.3; −1.6)	
		2017–2019	3.7 (−8.5; 17.6)	
Women				
0+	1	2000–2013	−1.9* (−2.9; −0.8)	−3.2* (−4.3; −2.0)
		2013–2019	−6.0* (−9.1; −2.7)	
5–14	0	2000–2019	2.0 (−2.8; 7.0)	
15–24	0	2000–2019	0.5 (−0.7; 1.6)	
25–44	0	2000–2019	−3.9* (−4.8; −3.0)	
45–64	1	2000–2013	−1.1 (−2.5; 0.3)	−3.6* (−5.1;-2.0)
		2013–2019	−8.7* (−12.8; −4.4)	
65+	1	2000–2009	−0.6 (−2.8; 1.7)	−2.9* (−4.2; −1.6)
		2009–2019	−5.0^a^ (−6.8; −3.1)	

*p < 0.05.

AAPC, Average Annual Percentage Change; APC, Annual Percentage Change; CI, Confidence Interval.

Among women, deaths by suicide decreased from 972 in the year 2000 to 610 in the year 2019, whereas SDR decreased from 5.2 to 3.0 per 100,000 women, respectively (Figure 1). AAPC over the entire 20-year period was −3.2% (*p* < 0.05), with the rate of decline being −1.9% (*p* < 0.05) between 2000 and 2013 and accelerating to −6.0% (*p* < 0.05) between 2013 and 2019 (Table 2).

Large differences were observed in SDR values and in trends of their changes between age groups. In the male group, the highest SDR values were found in the group aged between 45 and 64 years (Figure 2A). In 2000, SDR in this age group was 42.6 per 100,000. Between 2000 and 2012, SDR values increased at an average annual rate of 1.2% (*p* < 0.05). After 2012, they started to decrease (APC = −7.3%, *p* < 0.05), reaching a value of 29.6 in the year 2019 (Table 2). SDR in the second highest male group, *i.e.* among the respondents aged 65 years or above increased between 2000 and 2013 (APC = 0.7%, *p* < 0.05), however, between 2013 and 2017, this valued decreased (APC = −7.6%, *p* < 0.05). After 2017, SDR increased (APC = 3.7, *p* > 0.05) reaching a value of 29.0 in 2019. The male group, aged 25–44 years, demonstrated statistically significant decreases in SDR values between the years 2000 and 2007 (APC = −2.7%) as well as between the years 2012 and 2017 (APC = −6.9%). SDR values increased statistically insignificantly in the period 2007–2012 (APC = 2.1%) and 2017–2019 (APC = 4.8%). As a result of these changes, the SDR value decreased in this age group from 33.3 in 2000 to 24.1 in 2019. In the group of young men, aged 15–24 years, SDR values were stable between 2000 and 2014, ranging from 7.9 to 9.0 (APC = 0.0, *p* > 0.05). After 2014, SDRs began to decline at an average annual rate of −7.4% (*p* < 0.05), reaching a value of 6.1 in 2019.

Among women, as in the male group, the highest SDRs due to suicide were observed in the age group 45–64 years (Fiure. 2b). The SDR value was 8.1 in 2000 and 4.3 in 2019 per 100,000 females. AAPC over the entire analysed period was −3.6% (*p* < 0.05), with a small and statistically insignificant decrease between 2000 and 2013 (APC = −1.1%, *p* > 0.05). Between 2013 and 2019, it accelerated to −8.7% (*p* < 0.05) (Table 2). Among women aged 65 years or above, the SDR value decreased from 6.8 in 2000 to 4.0 in 2019. APC in the years 2000–2009 was −0.6% (*p* > 0.05), whereas in the years 2009–2019, it was −5.0% (*p* < 0.05).

For women aged 25–44 years, the SDR value decreased from 5.2 in 2000 to 2.6 in 2019 (APC = −3.9%, *p* < 0.05). A statistically insignificant increase in SDR, i.e., from a value of 1.3 in 2000 to a value of 1.5 in 2019 (APC = 0.5%, *p* > 0.05) was observed in the group of women aged 15–24 years.

In the group aged 5–14 years, 365 children died by committing suicide between the years 2000 and 2019. The number included 264 boys and 101 girls. In 2000, the number of deaths due to this cause was 34 (27 boys and 7 girls) and SDRs values were 0.38 and 0.10 per 100,000 population, respectively. In 2019, there were 16 deaths due to suicide (10 boys and 6 girls), whereas SDRs were 0.20 and 0.13.

### Suicide Odds According to Sociodemographic Characteristics

For each socio-demographic group, the odds of death due to suicide were calculated, and then using logistic regression, odds ratios (OR) were determined.

An analysis by age group showed that the chance of death by suicide was highest in the youngest age groups. The OR values for age groups 15–24 years vs. 65 years or above were 51.47 for males and 181.89 for females in 2019 (Table 3). Also, in the age groups 25–44 years and 45–64 years in both males and females, the chance of death by suicide was significantly higher than in the group aged 65 years or above. The OR values for age groups 25–44 vs. 65 years or above was 26.76 in the male group and 46.57 in the female group. For age groups 45–64 years vs. 65 or above, ORs were 5.18 in the male group and 9.50 in the female group.

**TABLE 3:** Suicide, odds ratios and 95% confidence intervals according to selected sociodemographic characteristics; Poland, 2000 and 2019.

Characteristics	Men						Women					
	2000			2019			2000			2019		
	OR	95% CI	*P*	OR	95% CI	*p*	OR	95% CI	*p*	OR	95% CI	*p*
Age group												
15–24	51.46	45.61–58.05	<0.001	51.47	44.23–59.91	<0.001	77.98	60.47–100.55	<0.001	181.89	133.84–247.18	<0.001
25–44	27.96	25.40–30.77	<0.001	26.76	24.46–29.27	<0.001	42.88	35.64–51.58	<0.001	46.57	37.28–58.17	<0.001
45–64	6.84	6.23–7.52	<0.001	5.18	4.75–5.65	<0.001	11.35	9.56–13.47	<0.001	9.50	7.78–11.59	<0.001
65+ (referent)	1.00			1.00			1.00			1.00		
Educational level												
Elementary	1.16	1.05–1.27	0.008	1.08	1.01–1.15	0.029	0.22	0.13–0.37	<0.001	0.25	0.19–0.32	<0.001
Secondary	2.20	1.99–2.41	<0.001	1.30	1.21–1.39	<0.001	0.85	0.47–1.54	>0.05	0.70	0.55–0.89	0.003
university (referent)	1.00			1.00			1.00			1.00		
Marital status												
Single	7.32	6.51–8.24	<0.001	8.22	7.17–9.42	<0.001	5.57	4.67–6.95	<0.001	12.35	9.75–15.64	<0.001
Married	2.14	1.92–2.40	<0.001	2.20	1.92–2.53	<0.001	5.58	4.74–6.57	<0.001	5.73	4.59–71,5	<0.001
Divorced	3.75	3.25–4.33	<0.001	3.71	3.18–4.33	<0.001	6.78	5.34–8.62	<0.001	4.32	3.21–5.82	<0.001
widowed (referent)	1.00			1.00			1.00			1.00		
Employment status^a^												
economically active	5.86	5.50–6.24	<0.001	5.46	5.14–5.78	<0.001	11.32	9.67–13.28	<0.001	11.80	10.07–13.83	<0.001
economically inactive (referent)	1.00			1.00			1.00			1.00		
Place of residence												
Rural	1.16	1.12–1.21	<0.001	1.60	1.50–1.70	<0.001	0.84	0.76–0.92	<0.001	1.15	1.02–1.28	0.008
urban (referent)	1.00			1.00			1.00			1.00		

^a^ Data on the employment status refer to the years 2000–2014. Since 2015, information on the employment status has been removed from death cards in Poland.

OR, Odds Ratios; CI, Confidence Interval.

An analysis of differences based on the education level showed that among men, the lowest chance of death by suicide was observed in the group with tertiary education. In 2019, the odds ratio was 8% higher in males with primary education, while males with secondary education demonstrated an OR value 30% higher than that observed among males with tertiary education. In the female group, an opposite trend was observed. The highest chance of death was noted for women with tertiary education. The odds ratio for women with secondary education was 30% lower in 2019. In the group of women with primary education, OR was 75% lower than in women with tertiary education.

A comparison by marital status showed that the lowest chance of death was observed for widows/widowers. In the group of unmarried women/unmarried men (single people), the OR value was 8.22 times higher in males and 12.35 times higher in females. The odds ratio in divorced males was 3.71 times higher than in widowers. Similarly, in divorced females, the OR value was 4.32 times higher than in widowed females. Also, in the group of married respondents, ORs were higher than in the group of widowed persons (2.20 for men and 5.73 for women).

While analysing the employment status of the deceased, the authors observed that the chance of death by suicide for economically active respondents was higher than in the economically inactive group. The odds ratios were 5.46 in the male group and 11.80 in the female group.

Men living in rural areas demonstrated a higher chance of committing suicide than those living in urban areas. In the year 2000, the odds ratio for rural residents was 16% higher than this parameter for urban residents. In 2019, the difference increased to 60%. In the year 2000, female rural residents demonstrated a 16% lower OR than female urban residents. In 2019, the situation was reversed - the OR value for women living in rural areas was 15% higher than for women living in urban areas.

### Analysis of Suicide Methods

Hanging is by far the most common suicide methods in both sexes and all age groups. Among men, it accounted for 91% of all suicides in 2000% and 89.8% in 2019 (Table 4). The second most frequent suicide method among men was jumping from height (2.2% in 2000% and 2.0% in 2019), and the third one was poisoning (with drugs - 1.4% and 1.2%, by other means - 1.2% and 3.4%). The methods observed in more than 1% also included cutting/piercing with a blunt object (1.1% and 1.5%) and using a firearm (0.7% and 1.4%).

**TABLE 4:** Suicide by method, gender and age group; Poland, 2000 and 2019.

Method	Age group											
	0+		5–14		15–24		25–44		45–64		65+	
	2000	2019	2000	2019	2000	2019	2000	2019	2000	2019	2000	2019
Men												
hanging (X70)	4430 (91.0%)	3553 (89.8%)	23 (85.2%)	9 (90.0%)	555 (89.1%)	253 (91.7%)	1610 (89.3%)	1272 (89.1%)	1716 (93.3%)	1316 (90.3%)	526 (91.0%)	703 (89.4%)
poisoning by drugs (X60-X64)	69 (1.4%)	49 (1.2%)	0 (0.0%)	0 (0.0%)	9 (1.4%)	5 (1.8%)	34 (1.9%)	28 (2.0%)	23 (1.3%)	8 (0.5%)	3 (0.5%)	8 (1.0%)
jumping from height (X80)	109 (2.2%)	80 (2.0%)	1 (3.7%)	1 (10.0%)	25 (4.0%)	9 (3.3%)	36 (2.0%)	37 (2.6%)	29 (1.6%)	14 (1.0%)	18 (3.1%)	19 (2.4%)
poisoning by other means (X65-X69)	57 (1.2%)	133 (3.4%)	0 (0.0%)	0 (0.0%)	8 (1.3%)	3 (1.1%)	22 (1.2%)	30 (2.1%)	24 (1.3%)	77 (5.3%)	3 (0.5%)	23 (2.9%)
firearms (X72-X74)	62 (1.3%)	49 (1.2%)	0 (0.0%)	0 (0.0%)	15 (2.4%)	2 (0.7%)	34 (1.9%)	20 (1.4%)	9 (0.5%)	16 (1.1%)	4 (0.7%)	11 (1.4%)
drowning (X71)	34 (0.7%)	7 (0.2%)	2 (7.4%)	0 (0.0%)	2 (0.3%)	0 (0.0%)	11 (0.6%)	2 (0.1%)	13 (0.7%)	4 (0.3%)	6 (1.0%)	1 (0.1%)
cutting/piercing with blunt object (X78)	53 (1.1%)	58 (1.5%)	0 (0.0%)	0 (0.0%)	0 (0.0%)	2 (0.7%)	26 (1.4%)	22 (1.5%)	15 (0.8%)	17 (1.2%)	12 (2.2%)	17 (2.2%)
Other	55 (1.1%)	28 (0.7%)	1 (3.7%)	0 (0.0%)	9 (1.5%)	2 (0.7%)	29 (1.6%)	16 (1.2%)	10 (0.5%)	4 (0.3%)	6 (1.0%)	4 (0.6%)
Women												
hanging (X70)	745 (76.6%)	493 (80.8%)	7 (100%)	6 (100%)	60 (63.2%)	55 (85.9%)	209 (75.7%)	116 (78.4%)	311 (79.3%)	178 (80.5%)	158 (78.2%)	138 (80.7%)
poisoning by drugs (X60-X64)	80 (8.2%)	48 (7.9%)	0 (0.0%)	0 (0.0%)	12 (12.6%)	5 (7.8%)	29 (10.5%)	17 (11.5%)	34 (8.7%)	17 (7.7%)	5 (2.5%)	9 (5.3%)
jumping from height (X80)	69 (7.1%)	36 (5.9%)	0 (0.0%)	0 (0.0%)	10 (10.5%)	2 (3.1%)	17 (6.2%)	7 (4.7%)	24 (6.1%)	12 (5.4%)	18 (8.9%)	15 (8.9%)
poisoning by other means (X65-X69)	12 (1.2%)	12 (2.0%)	0 (0.0%)	0 (0.0%)	1 (1.1%)	0 (0.0%)	5 (1.8%)	2 (1.4%)	4 (1.0%)	7 (3.2%)	2 (1.0%)	3 (1.8%)
drowning (X71)	31 (3.2%)	4 (0.7%)	0 (0.0%)	0 (0.0%)	3 (3.2%)	0 (0.0%)	8 (2.9%)	2 (1.4%)	10 (2.6%)	1 (0.4%)	10 (5.0%)	1 (0.5%)
cutting/piercing with blunt object (X78)	14 (1.4%)	11 (1.8%)	0 (0.0%)	0 (0.0%)	2 (2.1%)	1 (1.6%)	3 (1.1%)	1 (0.7%)	4 (1.0%)	5 (2.4%)	5 (2.5%)	4 (2.3%)
Other	21 (2.3%)	6 (0.9%)	0 (0.0%)	0 (0.0%)	7 (7.3%)	1 (1.6%)	5 (1.8%)	3 (1.9%)	5 (1.3%)	1 (0.4%)	4 (1.9%)	1 (0.5%)

Among women, hanging accounted for 76.6% of suicides in 2000 and it increased to 80.8% in 2019. However, the next most common three methods were used less frequently—poisoning with drugs (8.2% in 2000 and 7.9 in 2019), jumping from height (7.1% and 5.9%, respectively) and drowning (3.2% and 0.7%). The prevalence of poisoning by other means (1.2% and 2.0%) and cutting/piercing with a blunt object (1.4% and 1.8%) increased slightly.

## Discussion

According to WHO, in the 20-year period between 2000 and 2019, the global age-standardised suicide rate decreased by 36% worldwide and by 47% in the European region [1]. Our own study showed that SDR in Poland declined only by 28% over the same period (from 16.2 in 2000 to 11.7 in 2019 in total in both gender groups).

Positive changes in suicide mortality trends have been observed after 2012. To some extent, this may be related to the improvement of the economic situation in Poland. The unemployment rate fell from 13.4% in 2012% to 5.2% in 2019. The average monthly gross salary increased in this period from PLN 3,744 to PLN 5,182. Additionally, the extent of relative poverty decreased from 16.3% of people in households in 2012% to 13.0% in 2019. Extreme poverty, i.e. the subsistence minimum, concerned 6.8% of people in 2012, and 4.2% in 2019. Disproportions in the income of the Polish population also decreased in this period. The Gini coefficient dropped from 0.338 in 2012 to 0.301 in 2019 [16]. The observed favorable changes in suicide rates may also be related to the activities undertaken in Poland to improve the mental health of the population. In 2011, a National Mental Health Program for 2011–2015 was implemented in Poland [17]. In addition, since 2010, there has been implemented a Recommendation System for Prevention Programs and Mental Health Promotion in Poland. It is based on the criteria of the Exchange on Drug Demand Reduction Action (EDDRA) program realized by the National Bureau for Drug Prevention, the State Agency for Solving Alcohol-Related Problems, the Center of Education Development and the Institute of Psychiatry and Neurology [18]. In the studies of Polish authors, it was shown that between 2013 and 2018, the number of patients filling prescriptions for reimbursed antidepressants in Poland increased (from 948,400 to 1,277,900) with a simultaneous decrease in the number of suicides (from 6,101 to 5,182), noting a very high negative correlation between these two phenomena *(r* = −0.955; at *p* = 0.003). The number of patients filling prescriptions, however, may not reflect the number of adequately treated patients. Thus, the number of suicides per year was compared with the number (in millions) of daily doses (DDD, defined daily dose) of reimbursed antidepressants. The DDD number is a WHO-defined figure [19] and indicates the average daily dose for a drug used for its main indication in an adult on maintenance therapy [20]. These quantities also show a very high negative correlation (r = −0.959; at *p* = 0.002). The general positive changes observed in our study did not concern all the age groups. In the male group, the end of downward trend was observed in 2017 in groups aged 25–44 years and 65 years and above. There were not also positive changes throughout the analysed 20-year period in the group of the youngest women aged 5–24 years.

Various studies classify Poland as a country with strong gender-related determinants [4]. Globally, the age-standardised suicide rate was 2.3 times higher in males than in females. Male:female (M:F) suicide ratios above 1 indicate that suicide rates are higher in males than in females. While the ratio was slightly over 3 in high-income countries [1], in 2019 in Poland, it was 7.2. Some authors link the phenomenon of lower suicide rates in women to the protective function of motherhood, which is a barrier preventing women from engaging in self-injurious behaviour. Women are able to benefit more from social support and are less prone to aggressive behaviour and alcohol abuse [21]. On the other hand, the higher risk of suicide death among men is explained, among others, by the so-called “male depressive syndrome,” characterised by greater vulnerability to stress, reluctance to seek help, more frequent alcohol abuse [22]. However, these arguments can be applied to most societies, thus they do not explain the high suicide-related over-mortality of men in Poland.

Women more often use the so-called “soft” suicide methods, such as poisoning or drowning, which may be a reason for misclassification of the cause of death [2, 23]. Consequently, the number of female suicides is underestimated, which explains why there are so huge gender differences. Men are more likely to choose violent but effective suicide methods and these are easier to classify [24]. Rockett et al. in the study “Unrecognized self-injury mortality (SIM) trends among racial/ethnic minorities and women in the USA” extended the definition of suicide by 80% of “accidental” (“unintentional” under ICD-10 nomenclature) drug deaths (X40-44) and 90% of undetermined drug deaths (Y10-14) among persons aged >15 years [25]. After simulating these calculations for suicides in Poland in 2019, we found that the M:F suicide ratio decreased slightly from 7.2 to 6.6, still remaining at a very high level. The authors’ original study showed that about 90% of male suicides and about 80% of female suicides in Poland are committed by hanging. Although it is a common method of suicide in Europe, its prevalence in Poland is the highest one among European countries. To compare, the suicide death rate by hanging in Estonia was 79% for men and 71% for women, in Germany 55% for men and 38% for women, in Finland - 33% for men and 21% for women [26]. The prevalence of non-hanging suicides in Poland is very low, which implies that poisoning and other “soft” suicide methods may be underrepresented in Polish suicide statistics due to misclassification, which could partly explain high values of the male-to-female suicide ratio [7, 21].

For many years, the pattern of suicide mortality in Poland by age groups was typical of less developed countries, where suicides were mostly committed in the working age rather than in late old age. This pattern was common for Eastern Europe, and it was mainly observed in the former Soviet Union republics [27–29]. Our study revealed that in Poland the differences between the group aged 45–64 years and the group aged 65 years or above in both genders were gradually decreasing and in 2019, the values of suicide rates became very similar. If this trend continues in the following years, the pattern in Poland will resemble that characteristic for most European countries, such as Germany, Austria, Italy, Hungary, where suicide rates increase with age [26, 30, 31].

Suicide committed by young people is still a serious problem. Suicide is the second leading cause of death among young people aged 15–24 years in Europe, following unintentional injuries. A similar trend is observed in Poland. Among young men, values of suicide rates in 2000–2014 remained stable. After the year 2014, they began to decrease. In the group of young women, suicide rates are significantly lower than among men (M:F_2019_ = 4.1), however, an increasing trend was observed throughout the 20-year period (*p* > 0.05). It is also important to emphasize the problem of suicide in the group of children aged 5–14 years. The number of deaths due to suicide is small in this group, nevertheless, for social reasons they cannot be excluded from the analysis. In 2019 in Poland, SDR values in this age group were 0.20 for boys and 0.13 for girls.

Considering the general risk of mental health disorders in young people and the fact that previous suicide attempts lead to further ones [32], early provision of psychological or psychiatric counselling for people with depressive conditions and suicidal tendencies should be regarded as a priority, highlighted in the European youth health strategy [33]. Unfortunately, the situation in Poland in this area is poor. It is estimated that 9% of children and adolescents are seriously affected by mental disorders and they require professional assistance. It means that 630,000 children and adolescents under the age of 18 need psychiatric and psychological treatment [34]. According to WHO recommendations, in a middle-income country, the number of paediatric psychiatrists should be 10 per 100, 000 child and adolescent population [35]. Data obtained from the Supreme Audit Office show that in 2019 in Poland, there were 418 paediatric psychiatrists and the ratio was 6.0 per 100,000 population [36]. The shortage of medical staff is only one of the problems faced by paediatric psychiatry. Apart from that, there are also shortages of professional help for children with disruptive behaviour within social services and education, poor access to psychological help. Families of children with disorders also complain about an insufficient number of institutions offering support.

Results of many studies conducted around the world indicate that suicide mortality rates vary significantly depending on the level of education, marital status, employment status and place of residence [37–40]. The socioeconomic disadvantage is one of the major non-psychiatric determinants of suicidal behaviour. Being “socioeconomically disadvantaged” can be identified as living in relatively less favourable social and economic conditions than other individuals in the same society. Features of the socioeconomic disadvantage include low income, unmanageable debt, poor housing conditions, lack of educational qualifications, unemployment and living in a socioeconomically deprived area [41].

The risk of suicide is also increased by being widowed or divorced. A Polish study revealed that such individuals committed suicides four times more frequently than married or single men and women [42]. A sudden change in the marital status, caused by a divorce or death of a spouse/partner, evokes the feeling of loneliness, sense of isolation as well as social maladjustment. They all increase the risk of suicide.

The authors of this study used a slightly different method for analysing the relationship between sociodemographic variables and suicide rates. In order to compare particular groups, they calculated the probability of suicide death and odds ratios of death due to suicide in a given sociodemographic group. Application of this statistical method enabled them to show that young people (15–24 year-olds, followed by 25–44 year-olds), men with secondary education and women with tertiary education, married or single and economically active were burdened with the highest chance of dying due to suicide in 2019. Suicide deaths therefore predominate in groups of people with high social and economic potential.

Many studies also indicate that suicide rates were higher in rural areas than in urban areas, especially among men [43, 44]. Results of our study prove this thesis. This may be related to poorer access to psychiatric care [43] and higher alcohol consumption by rural residents [45]. Yet, in order to confirm such findings, a separate study is required.

The number of suicides is expected to increase significantly from 2020. The COVID-19 pandemic increased risk factors for suicide, such as economic downturn, poorer access to healthcare, deprioritisation of mental health and preventive activities, interpersonal conflicts, unemployment, poverty, loneliness and hopelessness. All these variables deepen depression, anxiety, post-traumatic stress disorder, contribute to harmful use of alcohol, substance abuse, and finally, increase the suicide risk [46].

### Conclusion

Despite decreasing trends in suicide rates occurring in the first 2 decades of the 21st century, Poland remains a country with a fairly high suicide risk.

An analysis of 20-year mortality trends in Poland reveals increasing suicide rates in the group of girls and young women aged 5–24 years. In the male groups aged 24–44 years and 65 years or above, growing suicide rates were observed after the year 2017. They require further observations.

What raises an alarming concern are an unusually high percentage of deaths due to hanging as well as the SDR value for male suicide, more than seven times higher than that for females, not observed in other countries.

There is a strong need to develop and implement psychological and educational programmes, primarily designed for young people. Such solutions might help them to cope with difficult situations and actively seek help.
